# The Motivational Aspect of Children’s Delayed Gratification: Values and Decision Making in Middle Childhood

**DOI:** 10.3389/fpsyg.2019.01649

**Published:** 2019-07-31

**Authors:** Louise Twito, Salomon Israel, Itamar Simonson, Ariel Knafo-Noam

**Affiliations:** ^1^Department of Psychology, The Hebrew University of Jerusalem, Jerusalem, Israel; ^2^School of Business, Stanford University, Stanford, CA, United States

**Keywords:** values, delay of gratification, children, behavior, motivation

## Abstract

Delayed gratification is the ability to postpone an immediate gain in favor of greater and later reward. Although delayed gratification has been studied extensively, little is known about the motivation behind children’s decisions. Since values are cognitive representations of individuals’ motivations, which serve to guide behavior, we studied the relationship between children’s values and delayed gratification. Two main distinct motivations overlapping with values may underlie this decision: conservation - the desire to reduce uncertainty and preserve the status quo, and self-enhancement – the desire to maximize resources and profit for the self. Accordingly, we hypothesized that conservation values would relate to children’s preference to hold on to what is given as soon as possible, and that self-enhancement values would relate to children’s preference for delaying gratification. Seven-year old children (*N* = 205) ranked their values with the Picture-Based Values Survey (Döring et al., 2010) as part of the Longitudinal Israeli Study of Twins (LIST) (Avinun and Knafo, 2013). The children also played a decision-making animation game that included delayed gratification decisions. In support of our hypotheses, greater delayed gratification related negatively to conservation values, specifically to security and tradition, and related positively to self-enhancement values, especially power and achievement. This is one of the first demonstrations that children’s values relate meaningfully to their behaviors.

## Introduction

One of the most important challenges we face in life is the need to delay gratification. The ability to either forgo immediate temptation or to persist in an undesirable activity, in order to reach a later goal, is a key component to success in many life tasks, such as preparing for exams (Bembenutty, 2007; Zhang et al., 2011), losing weight (Davis et al., 2004; Nederkoorn et al., 2006), quitting smoking (Bickel et al., 1999; Mitchell, 1999), and saving for retirement (O’Donoghue and Rabin, 2000; Wulfert et al., 2002). Delay of gratification is not a capability we are born with, but one achieved throughout development (Mittal et al., 2013). Therefore, it is an important aim to investigate delayed gratification in childhood and understand its underlying motivation. The current study aims to achieve this goal using data from a sample of children who reported their values and made economic decisions in which they were required to decide whether to delay or not to delay gratification.

### Delayed Gratification

Delayed gratification is the ability to postpone an immediate reward for the sake of more distant long-term gains. Generally, delayed gratification is assessed in tasks requiring individuals to forgo a smaller but immediate reward for the sake of receiving a larger reward in the future (Mischel and Ebbesen, 1970; Carducci, 2009).

The ability to delay gratification is known to increase with age (Mischel and Metzner, 1962; Yates et al., 1981; Thompson et al., 1997; Lemmon and Moore, 2007; Atance and Jackson, 2009). During the fourth year of life, children acquire the ability to deal with future-oriented situations (Thompson et al., 1997), and throughout the fifth-year, children exhibit cognitive strategies needed for delaying gratification (Mischel et al., 1989). Thus, by the age of five, children can opt to delay gratification (Moore and Macgillivray, 2004). Nevertheless, there are meaningful within-age individual differences. For example, in Carlson (2005) more than 30% of five-years-old children preferred NOT to delay gratification. Most of the extant research attributes this heterogeneity to individual differences in cognitive skills enabling delay, such as executive function (Baumeister and Vohs, 2003; Happaney et al., 2004; Zelazo and Carlson, 2012), and to variation in morphology and activation of brain regions sub-serving these capacities, such as the pre-frontal cortex (Mobini et al., 2002; McClure et al., 2004; Casey et al., 2011).

Although delay of gratification has been studied extensively, little is known about the motivational features underpinning children’s preference to delay gratification (Tobin and Graziano, 2009). Studies which deal with motivation typically relate to the situational constraints of the experiment. For example, children show greater motivation to refrain from eating a single cracker, if the delayed reward they can expect is larger (Carducci, 2009). Studies show that unreliable environments reduce children’s preference to wait for greater rewards. Delayed gratification requires trust in people who give the reward to deliver it in the future as promised, and if there is no trust, the child will prefer the certain option, an immediate if smaller gain (Kidd et al., 2013; McGuire and Kable, 2013; Michaelson et al., 2013).

Focusing on individual differences in the motivational aspects of delayed gratification, research has primarily focused on differences in personality traits underlying the successful implementation of self-control. For example, whether they have strong ego resiliency or strong willpower. Indeed, individuals who score higher on these traits tend to have more success in delaying gratification (Metcalfe and Mischel, 1999; Mischel and Ayduk, 2004). However, the content of this motivation, i.e., the personal motivational goals behind the desire to delay gratification, has not been studied. The motivation of some children for preferring the smaller reward has received even less attention. This leads to the question at the heart of the current investigation. What provides the motivation behind individuals’ decision to delay or not to delay gratification? And especially, what motivates some children to prefer a smaller reward?

To shed light on the motivational factors involved in delayed gratification, we propose to study the role of values, because values are cognitive representations of individuals’ motivations (Kluckhohn, 1951; Rokeach, 1973; Schwartz, 1992, 2012; Fischer and Boer, 2016). Values represent what is important or desirable, and thus suggest which behaviors should be performed (Miles, 2015). Indeed, values are systematically predictive of relevant behaviors (Sagiv and Roccas, 2017).

Therefore, we investigate the relationship between children’s values and decision making involving delayed gratification.

### Values

Values are defined as desirable, abstract goals, varying in importance across individuals and groups, and considered to be stable over time and across situations (Schwartz, 1992). Values serve as guiding principles for behavior, as principles to justify one’s behavior, and as guides toward the evaluation of other people and of the self (Rokeach, 1973; Schwartz, 1992; Schwartz et al., 2012). Thus, one of the reasons for values’ importance is their ability to influence and predict the individual’s perceptions, behavior and decision-making (Feather, 1995; Rohan, 2000; Roccas and Sagiv, 2010).

The most prominent framework for studying personal values in psychology is Schwartz’s theory of human values (Davidov et al., 2008). Schwartz (1992) identified ten universal personal value types, which differ in the type of motivational goals they express, specifically: universalism, benevolence, conformity, tradition, security, power, achievement, hedonism, stimulation, and self-direction.

According to Schwartz’s theory, there is a structure of dynamic relationships among values, representing the compatibilities and conflicts among them. The ten basic values are organized as a circular continuum according to the motivations they express, with adjacent values sharing compatible motivations and conflicting motivations reflected in opposite values (Davidov et al., 2008; Maio et al., 2009). The ten values can be also organized in four higher order values across two bipolar dimensions. The first dimension contrasts openness to change values (stimulation and self- direction) with conservation values (tradition, conformity, and security). The second represents self-transcendence values (universalism and benevolence) versus self-enhancement values (power and achievement). Hedonism shares element with openness to change and self-enhancement (Schwartz, 1992, 2012). Recent research demonstrates that this structure is replicated, using age-appropriate methods, already in middle childhood (Abramson et al., 2017; Collins et al., 2017).

Following up on value-behavior associations found in adults and adolescents (Roccas and Sagiv, 2010; Pulfrey and Butera, 2013; Benish-Weisman, 2015), a few recent studies conducted among children found value-behavior correlations.

Children’s values have been shown to relate meaningfully to behaviors reflective of the motivational aspect of each value (Vecchione et al., 2016). For example, children’s conservation values were reflected in their tendency to avoid dangers, while self-enhancement values related to behaviors such as doing one’s best to win competitions (Vecchione et al., 2016). In an experimental setting, children’s decision to donate candy to other children correlated positively with their relative preference for self-transcendence over self-enhancement values (Abramson et al., 2017). The ability of children to distinguish among values based on their motivational structure, and the relevance of values to children’s behavior, suggest the importance of values to children’s decisions. Below, we propose specific associations between values and delayed gratification.

### The Current Study

Values have been linked, theoretically and empirically, to motivational brain systems of approach and avoidance (Fischer and Boer, 2016; Fischer, 2017). Our hypotheses reflect such links between motivational systems and the values that promote them.

Gray’s reinforcement sensitivity theory (RST; Gray, 1970; Gray and McNaughton, 2000) a leading neurobiological theory of personality seeking to account for the underlying biological and psychological dynamics of individual differences (Fischer, 2017), focuses on two personality dimensions, anxiety and impulsivity. Each of these dimensions represents a different neurobiological system which responds uniquely to the environment. The first system, Behavioral Inhibition System (BIS) seeks to inhibit behavior which might have negative or painful outcomes. In other words, it is underlain by the motivation to prevent aversive stimuli. A person who has a sensitive BIS tends to anxiety reactions, avoidance and desire for security. Concerned with avoidance of novel and uncertain situations or stimuli, the BIS can be seen as represented by Conservation values (Fischer, 2017).

Another system described by RST is the Behavioral Activation System (BAS), responsible for motivation to reach pleasant stimuli. BIS and BAS are sensitive to different kind of reinforcements (Torrubia et al., 2001). While BIS is sensitive to negative reinforcement, BAS is sensitive to positive reinforcement (Carver and White, 1994). When a person has to make a decision to delay or not to delay gratification, a person with a sensitive BIS might want to avoid the risk of loss, leading to preference for a smaller and immediate gain. In contrast, a person who has a sensitive BAS would focus on the opportunity to get a larger gain and would prefer to delay gratification. The BAS, with its approach motivation which enables or promotes seeking opportunities and engaging in novel situations, can be seen as overlapping with Self-enhancement values (Fischer, 2017).

Thus, when children, or adults, deliberate whether to prefer a smaller but immediate reward or a larger reward in the future, two different motivations, overlapping with the core dimensions of values, may underlie their decision. The first dimension of values (Schwartz, 1992) contrasts conservation with openness to change. Conservation values, focusing on reducing uncertainty and preserving the status quo (Schwartz, 2012), were hypothesized to relate to children’s preference to hold on to what is given as soon as possible, because conservatives prefer the expected and known and avoid unpredictable situations (Schwartz et al., 2014), which is immanent in the choice to delay gratification. In contrast, openness to change values characterize individuals motivated to seek and accept change, innovation and adventure (Schwartz, 2012). We therefore expected children high in conservation values to be more likely to prefer a safe reward, if smaller, over an uncertain, larger reward.

The second dimension of values contrasts self-transcendence values of caring for others, with self-enhancement values, focusing on power and achievement. A focus on self-enhancement values is compatible with a motivation to attain resources for the self (Fischer, 2017), and we thus expected self-enhancement to relate positively to children’s preference for maximization of profit via delaying gratification. Based on the motivational structure of values (Schwartz, 2007) decisions correlating positively with one end of a value dimension would correlate negatively with the other end of the value dimension. This would mean a negative correlation between self-transcendence values and delayed gratification. On the other hand, because the cover story of the decisions children engaged in was to obtain rewards for a later prosocial behavior (as detailed in the method section), self-transcendence could also relate positively to delayed gratification in the current case. We therefore do not propose a hypothesis in the case of self-transcendence.

## Materials and Methods

### Participants

The sample includes 205 seven-year old children (mean age = 90.60 months, *SD* = 3.46), of whom 109 (53.1%) are boys. 91% of the families lived in two-parent households, and the total number of children averaged 3.69 (*SD* = 1.59). The participants were part of a larger sample, participating in the Longitudinal Israeli Study of Twins (LIST), in which all Hebrew-speaking families of twins born in Israel during 2004 – 2005 were invited to participate (Avinun and Knafo, 2013). When children reached the age of seven, the families living in Greater Jerusalem were invited to take part in an experimental session in the laboratory. Current participants are a subsample of the children who participated in a study of the genetics of values (Uzefovsky et al., 2016). The protocol for the experiment was approved by the Hebrew University Social Sciences research ethics committee, and written informed consent was obtained from participants’ parents.

### Measures

#### Values

Values were assessed with the Picture-based Value Survey for Children (Döring et al., 2010), which was specifically designed to study values in childhood. The tool has been translated to Hebrew and slightly adjusted to Israeli culture, as described by Abramson et al. (2017), and Uzefovsky et al. (2016).

The experimenter asked the children “How would you like to be in your future life.” Then, children were shown 20 cartoon-like pictures, two pictures for each value type. The pictures were printed on removable stickers. Each cartoon describes the same character performing a value-relevant action, accompanied by a brief caption. For example, the picture of a child wearing a helmet while riding the bicycle, and the text “to be safe” represent security values; an image of a winner on a podium accompanied by the caption “to be best” represent achievement values, and so on.

An experimenter presented the child with all of the items and read each caption out loud. Children were then asked to sort the items according to five levels of importance. First, the child was asked to choose two “Very important” items, then two “Not at all important” items. Then, the child was asked to choose four items as “important” and four as “not important.” Finally, the remaining eight items were automatically ranked in the intermediate level. Thus, the score in the scale ranges from 1 (not important at all) to 5 (very important). Structural analyses (reported by Uzefovsky et al., 2016) indicated that children’s value structure closely resembled the prototypical Schwartz (1992) value structure found with adults and adolescents.

The ranking scores for each pair of items measuring the same value were averaged to obtain a score on each of the 10 values. In addition, to reduce the number of analyses, values were aggregated to form higher-order value scores of self-transcendence (universalism and benevolence), conservation (conformity, tradition, and security), self-enhancement (power and achievement), and openness to change (stimulation, self-direction, and hedonism).

#### Delayed Gratification

**FIGURE 1:**
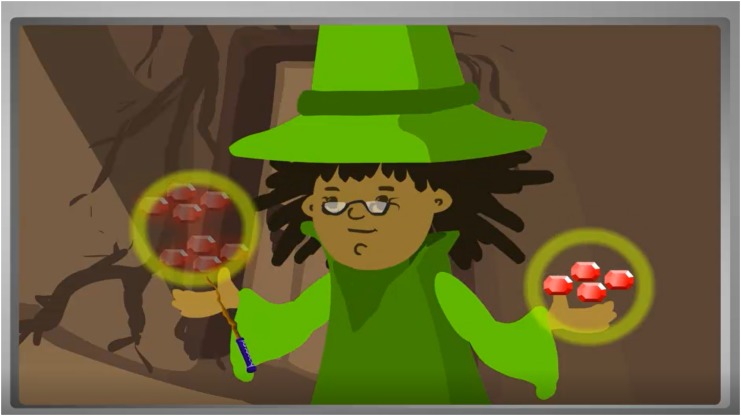
An example of one of the delayed gratification decisions. Children were asked to choose between receiving 4 gems immediately and receiving eight gems later.

Delayed Gratification was measured by an animation game, which was created especially for the current research. In the game, designed to engage children, the child was asked to help free the city by beating the evil wizard, who took all the colors from the city before the festival, and made everybody sad. To achieve this goal and free the city the child was required to collect as many gems as possible. Collecting and using gems along the way required making 23 decisions which simulated situations of economic gain or loss. During the game, among other decisions, there were three decisions that measured delayed gratification. In these decisions a witch, a lion, or a turtle offered the child the choice between a small, immediate reward, and a later but double in size, reward (5 or 10, 10 or 20, and 10 or 20, respectively, for the three trials). The time when the later reward would be given was unknown. See Figure 1 for an example.

The child’s choices (0 = immediate and small reward, 1 = delayed and larger reward) were summed into one variable labeled delayed gratification. The scale ranged from 0 to 3, based on the count of times in which a delayed reward was chosen.

#### Socioeconomic Status

As families’ socioeconomic status (SES) has been linked to both children’s values (Uzefovsky et al., 2016) and their delayed gratification (Green et al., 1996), we included SES in our design. SES was indicated in the current sample (Avinun and Knafo-Noam, 2017), by three mother-rated variables: household income (rated as much below, below, similar, above, or much above a given national average income), mother’s years of education, and the ratio of household number of rooms/residents. These components were standardized and an average was calculated to create the SES score.

## Results

About half (53.2%) of the children were willing to delay gratification in all three opportunities. A substantial proportion of children delayed only once (13.2%) or twice (20%), while 13.7% of the children did not delay gratification in any of the three opportunities.

Preliminary analyses showed that delayed gratification did not relate to age variation (in months), or twins’ zygosity. Delayed gratification showed an insignificant relationship with the SES composite score and (*beta* = −0.02, *ns*), as well as its three components (household income, *beta* = −0.08, mother’s years of education, *beta* = 0.07, and number of rooms/number of resident ratio, *beta* = −0.06, all *ns*). Finally, there were small sex differences in delayed gratification, such that boys delayed gratification slightly more times (*M* = 2.31, *SD* = 0.99) than girls (*M* = 1.91, *SD* = 1.16), *t* = 2.61, *p* = 0.01.

**FIGURE 2:**
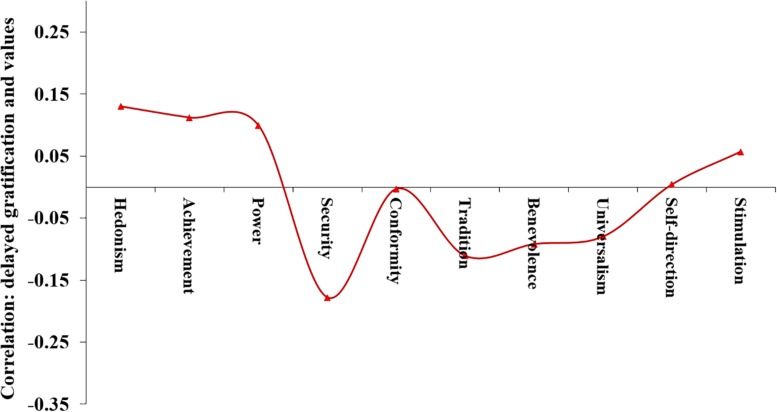
Correlations between delayed gratification and values.

Bivariate correlations between values and delayed gratification were computed using the TYPE = COMPLEX option in Mplus (Muthén and Muthén, 2010), which accounts for the non-independence of observations for individuals within the same family. In line with our hypothesis, delayed gratification related negatively to conservation values, *r* = −0.15, *p* = 0.018. Follow-up analysis showed that delayed gratification related specifically to security, and to a lesser extent tradition (see Figure 2). The motivationally opposed values of openness to change showed a modest, positive correlation with delayed gratification, *r* = 0.11, *n.s.*, reflecting a positive correlation with hedonism (Figure 2).

Moving on to the other major value dimension, delayed gratification related positively to self-enhancement values, *r* = 0.13, *p* = 0.035. We did not propose a directional hypothesis for the association between delayed gratification and the motivationally opposed values of self-transcendence. These values showed a negative correlation with delayed gratification, *r* = −0.11, *n.s*.

Because consistent (though small) sex differences have been observed in self-enhancement and self-transcendence values (Schwartz and Rubel, 2005; Knafo and Spinath, 2011), including in the current sample (Uzefovsky et al., 2016), we tested if sex accounted for the association between delayed gratification and self-enhancement. The association between self-enhancement values and delayed gratification was slightly reduced when sex was entered into the analysis as well, from β = 0.13 to β = 0.11, *p* = 0.06. Further analyses showed no interaction between sex and values in predicting delayed gratification.

**FIGURE 3:**
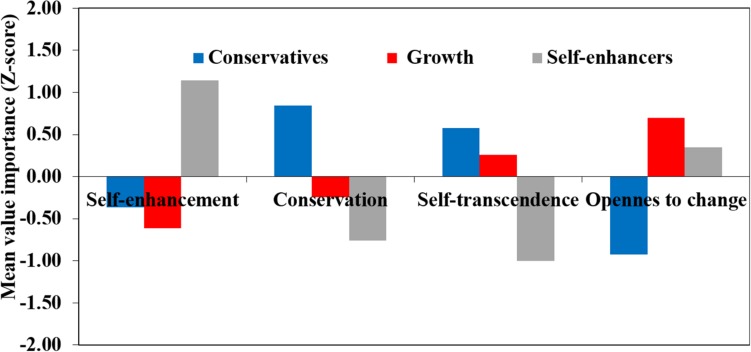
Means (*Z*-scores) of high-order value importance for each cluster.

To get a fuller understanding of our findings, we were interested in the role of specific combinations of values in children’s delayed gratification. Recent work has shown the advantage of using value profiles to understand their associations with other variables (Ungvary et al., 2018). We therefore assessed the association between delayed gratification and value profiles based on a cluster analysis, to examine how values work together beyond each value independently. In addition, we asked whether values related to the frequency of low delayed gratification or to that of high levels of delayed gratification. A 2-step cluster analysis was conducted, using data on all four higher-order values (an analysis using only self-enhancement and conservation yielded similar findings). The analysis identified three clusters, illustrated in Figure 3. The first cluster (29.8% of all participants) included children high on self-enhancement values and low on conservation values and self-transcendence. We refer to children in this cluster as “Self-enhancers” for simplicity.

The second cluster (33.7%) included children who were on average close to the mean on all four values, though higher on openness to change and self- transcendence than on conservation and self-enhancement. This distinction represents one organizing principle of the value system, contrasting values reflecting anxiety (conservation and self-enhancement, particularly power) with values reflecting growth (openness and self-transcendence, Schwartz, 2015). Therefore, we refer to this cluster as “Growth focus.” The third cluster (36.6%) was characterized by high degrees of conservation and low degrees of openness to change. We will refer to these children as “Conservatives.” As would be expected from the correlation analyses, self-enhancers showed the highest levels of delayed gratification (*M* = 2.33, *SD* = 0.81), and children with growth focus values were not very different on average (*M* = 2.17, *SD* = 1.14), while conservative children delayed gratification on average 1.92 times (*SD* = 1.23).

To understand whether values related to high or low levels of delayed gratification we next created two new measures for delay of gratification. The first distinguished children who chose a larger and later reward, in all three opportunities, from children who at least once chose not to delay gratification. The proportion of children choosing to delay gratification in all three opportunities did not differ across the three value clusters, χ^2^(DF = 2) = 2.70, *ns*.

**FIGURE 4:**
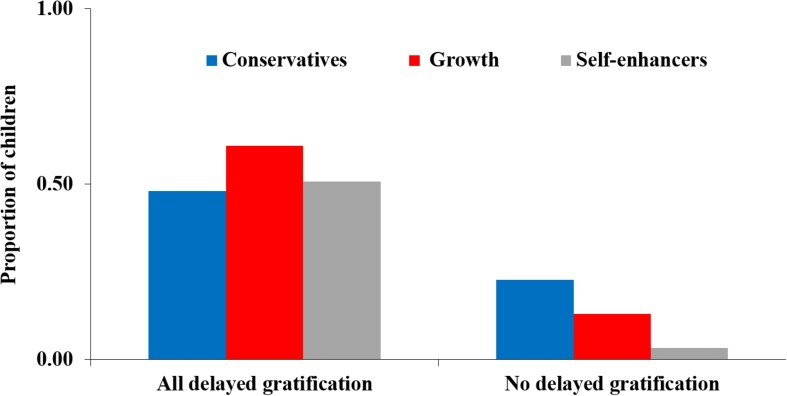
Proportion of children never delaying gratification across three decisions (left) and those always delaying gratification (right) by value clusters.

A different picture emerged when we compared the three value clusters in the prevalence of children who never chose to delay gratification. While almost a quarter (22.7%) of conservatives never delayed gratification, only two children (3.3%) in the self-enhancers group were not willing to delay gratification at least once, χ^2^(DF = 2) = 10.76, *p* = 0.005, *Cramer’s V* = 0.1619, a medium-size effect (see Figure 4).

## Discussion

Children’s tendency to delay gratification has been linked to long-term positive outcomes, including higher intelligence (Bembenutty and Karabenick, 2004; Duckworth and Seligman, 2005), social responsibility and social competence (Mischel et al., 1989), and improved academic performance (Mischel et al., 1988; Wulfert et al., 2002). Moreover, the ability to delay gratification has been identified as a protective factor against serious psychological and physical health problems, such as conduct disorders, antisocial behavior, hyperactivity, addictive problems (Mischel et al., 1989; Moffitt et al., 2011; Paulus et al., 2015) and obesity (Schlam et al., 2013). In addition, delayed gratification relates negatively to being convicted of a crime and having economic problems (Moffitt et al., 2011).

Despite the importance of research on delayed gratification, a gap regarding the content of the motivation underlying the decision to delay gratification exists. We addressed this question focusing on the relationship between 7-year-old children’s values and their decision-making involving delayed gratification. To the best of our knowledge, this is the first empirical demonstration of the association between delay of gratification and values. In support of our hypotheses, conservation values, especially security and tradition, correlated negatively, and self-enhancement values positively, with delayed gratification.

Children valuing conservation values prefer an immediate reward, even if it is smaller. As with the proverbial “bird in the hand,” individuals with high conservation values are more likely than individuals high on openness values to prefer the known and safe even if changes have the potential to lead to better outcomes (Gable et al., 2003; Janoff-Bulman, 2009). This is in line with main motivation underlying conservation values, especially security and tradition: preserving the status quo and avoiding unexpected situations (Schwartz, 2012; Schwartz et al., 2012). Interestingly, conformity values did not show the same association with delayed gratification as other conservation values did. In our previous work, in contexts involving trust in authorities, conformity did not relate to other variables in a way similar to the other conservation values (Nir and Knafo, 2009; Daniel et al., 2013). Possibly conformity values, which promote relying on social norms and authorities, are related to children’s likelihood of trusting the experiment and foregoing a current reward for a later reward, an effect opposed to the overall tendency of conservation values to avoid uncertainty.

In contrast, children focusing on self-enhancement prefer to wait and maximize rewards. This reflects the motivation underlying self-enhancement values, that is the desire to have more resources available to the individual and to increase one’s power and influence (Schwartz, 2012; Schwartz et al., 2012). The current work may be compared with work on Trinidadian adolescents (11–14-year-old), where individuals with a high need for achievement tended to prefer to wait until they achieved a higher reward (Mischel, 1961). While need for achievement, defined as “competition with a standard of excellence” (McClelland et al., 1953), is also conceptually related to self-direction values, it is possible that the self-enhancement aspect of achievement is responsible for the associations reported by Tobin and Graziano (2009). Future work should seek to investigate whether achievement values are involved in the longitudinal relationships between children’s delay of gratification and their future scholastic achievements (Mischel et al., 1989; Wulfert et al., 2002).

However, it is important to note, that the effect sizes of most findings are modest. All the betas we observed were between 0.13 and 0.19. Moreover, although this study refers to values as motivational factors for delaying or not delaying gratification, it is limited in its ability to detect the causal influence of values on behaviors. Experimental work suggest that values can have a causal influence on behavior (Maio et al., 2009; Sagiv and Roccas, 2017). Nevertheless, because values can also be used to justify one’s behavior (Schwartz, 1992) causal effects from behavior to values are also possible. We are aware of only one longitudinal study on children’s values and behaviors. In that study, values and behaviors showed reciprocal longitudinal effects (Vecchione et al., 2016). Future research would benefit from longitudinal designs which would allow for greater inference regarding the direction of causality between values and delay of gratification.

### Developmental Perspective

Until about a decade ago, there was a theoretical assumption that the development of values occurs in adolescence, with the formation of identity. However, with the recent development of research tools enabling the measurement of values among children (Döring et al., 2010; Collins et al., 2017), studies indicated meaningful value systems are present already in middle childhood, replicating the Schwartz (1992) value structure found in adolescents and adults (Cieciuch et al., 2016; Collins et al., 2017), even among children as young as 7 (Uzefovsky et al., 2016) and 5 years old (Abramson et al., 2017). This line of research opened ground for studies of the relations between values and behavior in children.

This is the first study of the relation between values and delayed gratification in childhood, which has the advantage of being a more informative period for assessing delay of gratification behavior. Additionally, this study is one of the first studies to show a relation between children’s values and behavior. The current findings are consistent with what has been found among adults and adolescents, that individuals’ values have a relationship with their behavior (Roccas and Sagiv, 2010; Pulfrey and Butera, 2013; Benish-Weisman, 2015).

Children’s preferred choice in their decision making in our study involved delay of gratification. Half of the children opted to wait for the larger reward and most of them decided to delay gratification at least one time (of 3 times). Based on past research showing that delayed gratification increases from early to middle childhood (Mischel and Metzner, 1962; Yates et al., 1981; Thompson et al., 1997), younger children are expected to show lower levels of delayed gratification, and larger variability. This larger variability, on the one hand, might enable finding stronger associations with values in younger children. On the other hand, there is some (limited) evidence that value-behavior relationships become stronger with age during childhood (Henshel, 1971; Abramson et al., 2017). Research on children’s values and behaviors from early to middle childhood is sorely needed.

### Limitations

Although correlations between delay of gratification and values were observed, the effects were small in magnitude and further research is needed to understand what moderates the association between values and delayed gratification. One potential reason for the low correlations is the low variability in delayed gratification, which could result from the age of the sample, as noted. Another possibility is that children were more likely to forego current rewards when they were presented in the context of a computerized game, than they would if presented with real-life rewards such as a marshmallow candy. Replication of the current findings with such rewards is an important direction for future research.

Moreover, due to the relatively small number of decision trials, our delay of gratification task limits our ability to compare our findings more generally to delay discounting tasks commonly used in experimental economics (Green et al., 1996; Green and Myerson, 2004; Odum, 2011). Such tasks typically present subjects with a series of choices in which they must indicate a preference between smaller and more immediate rewards (e.g., $10 now), vs. larger and more distant rewards (e.g., $100 in 1 year). Immediate or distant rewards are varied systematically (e.g., $20 now vs. $100 in 1 year) until subjects reach an indifference point; whereby the immediate and delayed quantities are of equivalent subjective value. These tasks allow for a more finely tuned parameterization of the features characterizing delay of gratification and have the potential to contribute to our understanding of the association between values and delay of gratification. In contrast, our study is different from past work as the participants were not told when they could expect the future reward, which may have introduced an additional element of uncertainty into participants’ decisions. We chose this more limited design in order to maximize 7 year old children’s engagement with the task. Future work on children may develop measures for assessing delayed discounting which will then be associated with children’s values.

Another potential limitation of our study is the composition of the sample: 7-year-old twins from the Jewish-Israeli population. Twins have not been shown to have substantially different personalities when compared to singletons (Johnson et al., 2002), but extending the findings with children from other cultures and religious backgrounds can elucidate important information about how the link between values and behavior may vary across different cultures. Finally, our twin sample was not large enough for complex genetic analyses. In a follow-up study with an expanded sample we intend to study the genetic and environmental contributions to children’s decision making in the context of delayed gratification, as well as the role of genes and the environment in the association of delayed gratification with values.

## Conclusion

The results support our hypothesized associations between children’s high self-enhancement and low conservation values and their tendency to delay gratification. Accordingly, the results suggest that potential gain and distrust in the ability to retain a future reward, respectively, promote or hinder delayed gratification. Following the same children through adolescence in additional developmental research, this study will have implications for understanding how the motivation to delay gratification develops, contributing to children’s and adolescents’ functioning.

## Ethics Statement

This study was approved by the Hebrew University Social Sciences Research Ethics Committee.

## Author Contributions

SI, IS, and AK-N designed the research. LT wrote the first draft of the manuscript. LT and AK-N were responsible for the analyses. All authors made contributions to the conception of the manuscript and participated in the writing process by adding substantively relevant content.

## Conflict of Interest Statement

The authors declare that the research was conducted in the absence of any commercial or financial relationships that could be construed as a potential conflict of interest.

## References

[B1] Abramson L. Daniel E. Knafo-Noam A. (2017). The role of personal values in children’s costly sharing and non-costly giving. *J. Exp. Child Psychol.* 165 117–134. 10.1016/j.jecp.2017.03.007 28442138

[B2] Atance C. M. Jackson L. K. (2009). The development and coherence of future-oriented behaviors during the preschool years. *J. Exp. Child Psychol.* 102 379–391. 10.1016/j.jecp.2009.01.001 19232416

[B3] Avinun R. Knafo A. (2013). The Longitudinal Israeli study of twins (LIST)—an integrative view of social development. *Twin Res. Hum. Genet.* 16 197–201. 10.1017/thg.2012.73 23394191

[B4] Avinun R. Knafo-Noam A. (2017). Parental brain-derived neurotrophic factor genotype, child prosociality, and their interaction as predictors of parents’ warmth. *Brain Behav.* 7:e00685. 10.1002/brb3.685 28523227 PMC5434190

[B5] Baumeister R. F. Vohs K. D. (2003). Self-regulation and the executive function of the self. *Handbook Self Ident.* 1 197–217.

[B6] Bembenutty H. (2007). Self-regulation of learning and academic delay of gratification: gender and ethnic differences among college students. *J. Adv. Acad.* 18 586–616. 10.4219/jaa-2007-553

[B7] Bembenutty H. Karabenick S. A. (2004). Inherent association between academic delay of gratification, future time perspective, and self-regulated learning. *Educ. Psychol. Rev.* 16 35–57. 10.1023/b:edpr.0000012344.34008.5c

[B8] Benish-Weisman M. (2015). The interplay between values and aggression in adolescence: a longitudinal study. *Dev. Psychol.* 51:677. 10.1037/dev0000015 25844848

[B9] Bickel W. K. Odum A. L. Madden G. J. (1999). Impulsivity and cigarette smoking: delay discounting in current, never, and ex-smokers. *Psychopharmacology* 146 447–454. 10.1007/pl00005490 10550495

[B10] Carducci B. J. (2009). *Basic processes of Mischel’s Cognitive-Affective Perspective: Delay of Gratification and Conditions of Behavioral Consistency The Psychology of Personality: Viewpoints, Research, and Applications.* Hoboken: John Wiley and Sons, 338–346.

[B11] Carlson S. M. (2005). Developmentally sensitive measures of executive function in preschool children. *Dev. Neuropsychol.* 28 595–616. 10.1207/s15326942dn2802_3 16144429

[B12] Carver C. S. White T. L. (1994). Behavioral inhibition, behavioral activation, and affective responses to impending reward and punishment: the BIS/BAS scales. *J. Person. Soc. Psychol.* 67:319 10.1037//0022-3514.67.2.319

[B13] Casey B. J. Somerville L. H. Gotlib I. H. Ayduk O. Franklin N. T. Askren M. K. (2011). Behavioral and neural correlates of delay of gratification 40 years later. *Proc. Natl. Acad. Sci.* 108 14998–15003. 10.1073/pnas.1108561108 21876169 PMC3169162

[B14] Cieciuch J. Davidov E. Algesheimer R. (2016). The stability and change of value structure and priorities in childhood: a longitudinal study. *Soc. Dev.* 25 503–527. 10.1111/sode.12147

[B15] Collins P. R. Lee J. A. Sneddon J. N. Döring A. K. (2017). Examining the consistency and coherence of values in young children using a new Animated Values Instrument. *Person. Individ. Differ.* 104 279–285. 10.1016/j.paid.2016.08.024

[B16] Daniel E. Fortuna K. Thrun K. S. Cioban S. Knafo A. (2013). Brief report: early adolescents’ value development at war time. *J. Adoles.* 36 651–655. 10.1016/j.adolescence.2013.03.009 23849659

[B17] Davidov E. Schmidt P. Schwartz S. H. (2008). Bringing values back in: the adequacy of the European Social Survey to measure values in 20 countries. *Public Opin. Q.* 72 420–445. 10.1093/poq/nfn035

[B18] Davis C. Levitan R. D. Muglia P. Bewell C. Kennedy J. L. (2004). Decision-making deficits and overeating: a risk model for obesity. *Obesity* 12 929–935. 10.1038/oby.2004.113 15229331

[B19] Döring A. K. Blauensteiner A. Aryus K. Drögekamp L. Bilsky W. (2010). Assessing values at an early age: the picture-based value survey for children (PBVS–C). *J. Person. Assess.* 92 439–448. 10.1080/00223891.2010.497423 20706930

[B20] Duckworth A. L. Seligman M. E. (2005). Self-discipline outdoes IQ in predicting academic performance of adolescents. *Psychol. Sci.* 16 939–944. 10.1111/j.1467-9280.2005.01641.x 16313657

[B21] Feather N. T. (1995). Values, valences, and choice: the influences of values on the perceived attractiveness and choice of alternatives. *J. Person. Soc. Psychol.* 68:1135 10.1037//0022-3514.68.6.1135

[B22] Fischer R. (2017). *Personality, Values, Culture An Evolutionary Approach.* Cambridge: Cambridge University Press.

[B23] Fischer R. Boer D. (2016). Values: the dynamic nexus between biology, ecology and culture. *Curr. Opin. Psychol.* 8 155–160. 10.1016/j.copsyc.2015.12.009 29506792

[B24] Gable S. L. Reis H. T. Elliot A. J. (2003). Evidence for bivariate systems: an empirical test of appetition and aversion across domains. *J. Res. Person.* 37 349–372. 10.1016/s0092-6566(02)00580-9

[B25] Gray J. A. (1970). The psychophysiological basis of introversion-extraversion. *Behav. Res. Ther.* 8 249–266. 10.1016/0005-7967(70)90069-0 5470377

[B26] Gray J. A. McNaughton N. (2000). *The Neuropsychology of Anxiety: An Enquiry into the Function of the Septo-Hippocampal System*, 2nd Edn. Oxford: Oxford University Press.

[B27] Green L. Myerson J. (2004). A discounting framework for choice with delayed and probabilistic rewards. *Psychol. Bull.* 130 769–792. 10.1037/0033-2909.130.5.769 15367080 PMC1382186

[B28] Green L. Myerson J. Lichtman D. Rosen S. Fry A. (1996). Temporal discounting in choice between delayed rewards: the role of age and income. *Psychol. Aging* 11 79–84. 10.1037//0882-7974.11.1.79 8726373

[B29] Happaney K. Zelazo P. D. Stuss D. T. (2004). Development of orbitofrontal function: current themes and future directions. *Brain Cogn.* 55 1–10. 10.1016/j.bandc.2004.01.001 15134839

[B30] Henshel A. M. (1971). The relationship between values and behavior: a developmental hypothesis. *Child Dev.* 42:1997 10.1111/j.1467-8624.1971.tb03787.x 5146026

[B31] Janoff-Bulman R. (2009). To provide or protect: motivational bases of political liberalism and conservatism. *Psychol. Inquiry* 20 120–128. 10.1080/10478400903028581

[B32] Johnson W. Krueger R. F. Bouchard T. J. McGue M. (2002). The personalities of twins: just ordinary folks. *Twin Res. Hum. Genet.* 5 125–131. 10.1375/1369052022992 11931690

[B33] Kidd C. Palmeri H. Aslin R. N. (2013). Rational snacking: young children’s decision-making on the marshmallow task is moderated by beliefs about environmental reliability. *Cognition* 126 109–114. 10.1016/j.cognition.2012.08.004 23063236 PMC3730121

[B34] Kluckhohn C. (1951). “Values and value-orientations in the theory of action: an exploration in definition and classification,” in *Toward a General Theory of Action*, eds Parsons T. Shils E. (Cambridge, MA: Harvard University Press), 388–433.

[B35] Knafo A. Spinath F. M. (2011). Genetic and environmental influences on girls’ and boys’ gender-typed and gender-neutral values. *Dev. Psychol.* 47 726–731. 10.1037/a0021910 21142356

[B36] Lemmon K. Moore C. (2007). The development of prudence in the face of varying future rewards. *Dev. Sci.* 10 502–511. 10.1111/j.1467-7687.2007.00603.x 17552939

[B37] Maio G. R. Pakizeh A. Cheung W. Y. Rees K. J. (2009). Changing, priming, and acting on values: effects via motivational relations in a circular model. *J. Person. Soc. Psychol.* 97:699. 10.1037/a0016420 19785487

[B38] McClelland D. C. Atkinson J. W. Clark R. A. Lowell E. L. (1953). “The measuring instrument,” in *The achievement motive*, (East Norwalk, CT: Appleton-Century-Crofts), 185–217.

[B39] McClure S. M. Laibson D. I. Loewenstein G. Cohen J. D. (2004). Separate neural systems value immediate and delayed monetary rewards. *Science* 306 503–507. 10.1126/science.1100907 15486304

[B40] McGuire J. T. Kable J. W. (2013). Rational temporal predictions can underlie apparent failures to delay gratification. *Psychol. Rev.* 120:395. 10.1037/a0031910 23458085 PMC3773987

[B41] Metcalfe J. Mischel W. (1999). A hot/cool-system analysis of delay of gratification: dynamics of willpower. *Psychol. Rev.* 106:3. 10.1037//0033-295x.106.1.3 10197361

[B42] Michaelson L. de la Vega A. Chatham C. Munakata Y. (2013). Delaying gratification depends on social trust. *Front. Psychol.* 4:355. 10.3389/fpsyg.2013.00355 23801977 PMC3685794

[B43] Miles L. D. (2015). *Techniques of Value Analysis and Engineering.* Nebraska: Miles Value Foundation.

[B44] Mischel W. (1961). Delay of gratification, need for achievement, and acquiescence in another culture. *J. Abnor. Soc. Psychol.* 62:543 10.1037/h0039842 14474527

[B45] Mischel W. Ayduk O. (2004). “Willpower in a cognitive-affective processing system,” in *Handbook of Self-Regulation Research, Theory, and Applications*, eds Baumeister R. F. Vohs K. D. (New York, NY: The Guilford Press), 99–129.

[B46] Mischel W. Ebbesen E. B. (1970). Attention in delay of gratification. *J. Person. Soc. Psychol.* 16:329. 10.1037/h0032198 5010404

[B47] Mischel W. Metzner R. (1962). Preference for delayed reward as a function of age, intelligence, and length of delay interval. *J. Person. Soc. Psychol.* 64:425 10.1037/h0045046 14474526

[B48] Mischel W. Shoda Y. Peake P. K. (1988). The nature of adolescent competencies predicted by preschool delay of gratification. *J. Person. Soc. Psychol.* 54 687–696. 10.1037//0022-3514.54.4.687 3367285

[B49] Mischel W. Shoda Y. Rodriguez M. I. (1989). Delay of gratification in children. *Science* 244 933–938. 10.1126/science.2658056 2658056

[B50] Mitchell S. H. (1999). Measures of impulsivity in cigarette smokers and non-smokers. *Psychopharmacology* 146 455–464. 10.1007/pl00005491 10550496

[B51] Mittal R. Russell B. S. Britner P. A. Peake P. K. (2013). Delay of gratification in two-and three-year-olds: associations with attachment, personality, and temperament. *J. Child Family Stud.* 22 479–489. 10.1007/s10826-012-9600-6

[B52] Mobini S. Body S. Ho M.-Y. Bradshaw C. Szabadi E. Deakin J. (2002). Effects of lesions of the orbitofrontal cortex on sensitivity to delayed and probabilistic reinforcement. *Psychopharmacology* 160 290–298. 10.1007/s00213-001-0983-0 11889498

[B53] Moffitt T. E. Arseneault L. Belsky D. Dickson N. Hancox R. J. Harrington H. (2011). A gradient of childhood self-control predicts health, wealth, and public safety. *Proc. Natl. Acad. Sci. U.S.A.* 108 2693–2698. 10.1073/pnas.1010076108 21262822 PMC3041102

[B54] Moore C. Macgillivray S. (2004). Altruism, prudence, and theory of mind in preschoolers. *New Direct. Child Adoles. Dev.* 2004 51–62. 10.1002/cd.97 15112535

[B55] Muthén L. K. Muthén B. O. (2010). *Mplus: Statistical Analysis with Latent Variables: User’s Guide.* Los Angeles, CA: Muthén & Muthén.

[B56] Nederkoorn C. Braet C. Van Eijs Y. Tanghe A. Jansen A. (2006). Why obese children cannot resist food: the role of impulsivity. *Eat. Behav.* 7 315–322. 10.1016/j.eatbeh.2005.11.005 17056407

[B57] Nir L. Knafo A. (2009). Reason within passion: values as motivational anchors of Israeli Opinion on the 2006 Lebanon war and ceasefire. *Ann. N. York Acad. Sci.* 1167 146–157. 10.1111/j.1749-6632.2009.04600.x 19580561

[B58] O’Donoghue T. Rabin M. (2000). The economics of immediate gratification. *J. Behav. Decis. Making* 13:233 10.1002/(sici)1099-0771(200004/06)13:2<233::aid-bdm325>3.0.co;2-u

[B59] Odum A. L. (2011). Delay discounting: I’m ak, you’re ak. *J. Exp. Anal. Behav.* 96 427–439. 22084499 10.1901/jeab.2011.96-423 PMC3213005

[B60] Paulus M. Licata M. Kristen S. Thoermer C. Woodward A. Sodian B. (2015). Social understanding and self-regulation predict pre-schoolers’ sharing with friends and disliked peers: a longitudinal study. *Int. J. Behav. Dev.* 39 53–64. 10.1177/0165025414537923

[B61] Pulfrey C. Butera F. (2013). Why neoliberal values of self-enhancement lead to cheating in higher education: a motivational account. *Psychol. Sci.* 24 2153–2162. 10.1177/0956797613487221 24058068

[B62] Roccas S. Sagiv L. (2010). Personal values and behavior: taking the cultural context into account. *Soc. Person. Psychol. Compass* 4 30–41. 10.1111/j.1751-9004.2009.00234.x

[B63] Rohan M. J. (2000). A rose by any name? *Values Construct. Person. Soc. Psychol. Rev.* 4 255–277.

[B64] Rokeach M. (1973). *The Nature of Human Values.* New York, NY: Free press.

[B65] Sagiv L. Roccas S. (2017). *What Personal Values are and what they are not: Taking a cross-Cultural Perspective Values and Behavior.* Cham: Springer, 3–13.

[B66] Schlam T. R. Wilson N. L. Shoda Y. Mischel W. Ayduk O. (2013). Peschoolers’ delay of gratification predicts their body mass 30 years later. *J. Pediatr.* 162 90–93. 10.1016/j.jpeds.2012.06.049 22906511 PMC3504645

[B67] Schwartz S. H. (1992). Universals in the content and structure of values: theoretical advances and empirical tests in 20 countries. *Adv. Exp. Soc. Psychol.* 25 1–65. 10.1016/s0065-2601(08)60281-6 17874285

[B68] Schwartz S. H. (2007). Universalism values and the inclusiveness of our moral universe. *J. Cross Cult. Psychol.* 38 711–728. 10.1177/0022022107308992

[B69] Schwartz S. H. (2012). An overview of the Schwartz theory of basic values. *Online Read. Psychol. Cult.* 2:11 10.1017/cbo9780511805769.004

[B70] Schwartz S. H. (2015). “Basic individual values: sources and consequences,” in *Handbook of Value Perspectives from Economics, Neuroscience, Philosophy, Psychology and Sociology*, eds Brosch T. Sander D. (New York, NY: Oxford University Press), 63–84. 10.1093/acprof:oso/9780198716600.003.0004

[B71] Schwartz S. H. Caprara G. V. Vecchione M. Bain P. Bianchi G. Caprara M. G. (2014). Basic personal values underlie and give coherence to political values: a cross national study in 15 countries. *Polit. Behav.* 36 899–930. 10.1007/s11109-013-9255-z

[B72] Schwartz S. H. Cieciuch J. Vecchione M. Davidov E. Fischer R. Beierlein C. (2012). Refining the theory of basic individual values. *J. Person. Soc. Psychol.* 103:663. 10.1037/a0029393 22823292

[B73] Schwartz S. H. Rubel T. (2005). Sex differences in value priorities: cross-cultural and multimethod studies. *J. Person. Soc. Psychol.* 89:1010. 10.1037/0022-3514.89.6.1010 16393031

[B74] Thompson C. Barresi J. Moore C. (1997). The development of future-oriented prudence and altruism in preschoolers. *Cogn. Dev.* 12 199–212. 10.1016/s0885-2014(97)90013-7

[B75] Tobin R. M. Graziano W. G. (2009). “A review of fifty years of regulation research,” in *Handbook of Personality and Self-Regulation*, ed. Hoyle R. H. (Hoboken: Wiley-Blackwell), 47.

[B76] Torrubia R. Avila C. Moltó J. Caseras X. (2001). The sensitivity to punishment and sensitivity to reward questionnaire (SPSRQ) as a measure of Gray’s anxiety and impulsivity dimensions. *Person. Individ. Differ.* 31 837–862. 10.1093/scan/nsz011 30753654 PMC6399605

[B77] Ungvary S. McDonald K. L. Benish-Weisman M. (2018). Identifying and distinguishing value profiles in American and Israeli adolescents. *J. Res. Adoles.* 28 294–309. 10.1111/jora.12330 28653451

[B78] Uzefovsky F. Döring A. K. Knafo-Noam A. (2016). Values in middle childhood: social and genetic contributions. *Soc. Dev.* 25 482–502. 10.1111/sode.12155 16260716

[B79] Vecchione M. Döring A. K. Alessandri G. Marsicano G. Bardi A. (2016). Reciprocal relations across time between basic values and value-expressive behaviors: a longitudinal study among children. *Soc. Dev.* 25 528–547. 10.1111/sode.12152

[B80] Wulfert E. Block J. A. Santa Ana E. Rodriguez M. L. Colsman M. (2002). Delay of gratification: impulsive choices and problem behaviors in early and late adolescence. *J. Person.* 70 533–552. 10.1111/1467-6494.05013 12095190

[B81] Yates G. C. Lippett R. M. K. Yates S. M. (1981). The effects of age, positive affect induction, and instructions on children’s delay of gratification. *J. Exp. Child Psychol.* 32 169–180. 10.1016/0022-0965(81)90101-6

[B82] Zelazo P. D. Carlson S. M. (2012). Hot and cool executive function in childhood and adolescence: development and plasticity. *Child Dev. Persp.* 6 354–360.

[B83] Zhang L. Karabenick S. A. Maruno S. I. Lauermann F. (2011). Academic delay of gratification and children’s study time allocation as a function of proximity to consequential academic goals. *Lear. Instruct.* 21 77–94. 10.1016/j.learninstruc.2009.11.003

